# Duboisia and Atropia

**Published:** 1880-03-20

**Authors:** 


### *ACTION OF DUBOISIA AND ATROPIA.*

Dr. Sidney Ringer, in the *Practitioner*, says:

"Duboisia possesses the same properties as atropia, but is far more powerful than atropia. Mr. Tweedy found this to be the case in regard to the local application to the eye. But while duboisia is far more powerful than atropia on man, the reverse is the case in respect to frogs. Atropia paralyzes far more powerfully the motor nervous system, the heart and respirations, in frogs, than duboisia."

